# Postoperative course and prognostic value of circulating angiogenic cytokines after pancreatic cancer resection

**DOI:** 10.18632/oncotarget.20315

**Published:** 2017-08-17

**Authors:** Cui Yang, Ulrich Bork, Sebastian Schölch, Yakup Kulu, Lars Kaderali, Uta L. Bolstorff, Christoph Kahlert, Jürgen Weitz, Nuh N. Rahbari, Christoph Reissfelder

**Affiliations:** ^1^ Department of Gastrointestinal, Thoracic and Vascular Surgery, University Hospital Carl Gustav Carus, Technische Universität Dresden, Dresden, Germany; ^2^ Department of General, Gastrointestinal and Transplantation Surgery, University of Heidelberg, Heidelberg, Germany; ^3^ Institute of Bioinformatics, University of Greifswald, Greifswald, Germany

**Keywords:** pancreatic cancer, circulating cytokines, postoperative, VEGF, TNF-α

## Abstract

**Background:**

Circulating angiogenic cytokines (CACs) have been confirmed as prognostic biomarkers and therapeutic targets in several solid tumors. However, their role as prognostic biomarkers in resected pancreatic ductal adenocarcinoma (PDAC) is unknown.

**Results:**

The expression of CACs in patients with PDAC differs from those with CP both pre- and postoperatively. Correlation analyses show significant correlations between circulating levels of CACs: VEGF was correlated with IL-6 (*r* = 0.457), FGF (*r* = 0.44), G-CSF (*r* = 0.543), HGF (*r* = 0.586) and SDF-1α (*r* = 0.784) before the surgery. The circulating levels of TNF-α correlated with the serum concentration of IL-4 before (r= 0.656) and after the resection (*r* = 0.776 on POD 3, *r* = 0.865 on POD 7). Gender did not show any correlation with serum levels of CAC, except for significantly higher levels of EGF in males (*P* = 0.002). Other clinicopathological variables such as age (< 65 vs. > 65 years), T, N, or UICC stage did not have an association with the cytokine levels. The multivariate model including the entire angiogenic panel revealed that postoperative increasing levels of EGF (*P* = 0.023), PDGFA-A (*P* = 0.024), TNF-α (*P* = 0.001) and IL-8 (*P* = 0.049) were associated with a favorable prognosis, whereas elevating levels of VEGF (*P* = 0.005) correlated with a poor cancer-specific survival.

**Materials and Methods:**

Preoperative and postoperative blood samples were collected in patients undergoing surgery for PDAC (*n* = 40) or chronic pancreatitis (CP; *n* = 9). Serum levels of 13 angiogenic cytokines (IL-4, IL-6, FGF-b, G-CSF, TNF-α, VEGF, HGF, SDF-1α, IL-8, EGF, Ang-1, PDGF-AA and PlGF) were analyzed using ELISA and Multiplex. Prognostic factors were identified by a Cox proportional hazards model.

**Conclusions:**

Postoperative changes of serum levels of certain angiogenic cytokines correlate with patients’ prognosis after resection for pancreatic cancer. CACs should thus be considered as biomarkers in patients with resected pancreatic cancer.

## INTRODUCTION

In 2017, it is estimated that about 53670 new cases of pancreatic cancer will occur in the United States, making it currently the tenth most common solid malignancy; yet it is the fourth leading cause of cancer-related deaths in both sexes [1]. The overall 5-year survival rate is less than 5 %. Being the only potentially curative treatment, complete resection in combination with adjuvant chemotherapy may merely improve 5-year survival to about 20% [2, 3]. This poor outcome is linked to the fact that the disease is often detected in an advanced state, making a cure by surgical resection difficult [4–6].

The majority of patients die of tumor progression and ultimately metastatic disease. During tumor progression and metastasis, an ‘angiogenic switch’ is almost always present, providing the growing tumor mass with new vasculature for sufficient supply of oxygen and nutrients [7]. Angiogenesis within solid tumors is a complex process, involving numerous angiogenic factors which are produced by cancer cells themselves as well as by the tumor microenvironment [8]. Since the close relation of tumor angiogenesis and tumor progression became a well-established aspect in cancer biology [9], angiogenic cytokines have been studied intensively as prognostic biomarkers [10–12] and therapeutic targets [13, 14]. Several studies demonstrated circulating angiogenic cytokines (CACs) as prognostic biomarkers in patients with various solid tumors [11, 15]. Most pancreatic cancers display overexpression of key mediators of tumor angiogenesis, including the vascular endothelial growth factor (VEGF), tumor necrosis factor -α (TNF-α) and placental growth factor (PlGF) [16–18]. Most anti-angiogenic therapies developed to date have been directed towards specific ligands (e.g. VEGF) or their receptors. Nonetheless, resulting clinical responses have been disappointing [8, 19, 20]. One possible explanation is that angiogenesis may be regulated by redundant pathways. Subsequently, inhibition of one pathway may stimulate alternative signaling pathways and may not completely shut off the process of angiogenesis [8, 21].

In this study, we investigated the expression of 13 CACs in patients with resected pancreatic cancer and compared their CAC levels to the angiogenic profile of patients undergoing surgery for chronic pancreatitis. Furthermore, we evaluated the prognostic significance of these CACs in patients with resected pancreatic cancer. As a novel approach to considering the redundant pathways in cancer angiogenesis, a panel of 13 CACs were analyzed simultaneously.

## RESULTS

### Patient characteristics

The study population of 49 patients included 31 males (63.2%) and 18 females (36.8%) with a median age of 64.5 years (range 41– 80 years). PDAC was diagnosed in 40 patients (81.6%) who underwent pancreatic resection. 9 patients (18.4%) with a chronic pancreatitis served as a control group (Table 1).

**Table 1:** Clinicopathologic characteristics of the study population (*n* = 49)

	*N* (%), median (range)
Gender	
Male	31 (63.2)
Female	18 (36.8)
Age (Year)	64.5 (41 – 80)
Pancreatic adenocarcinoma	40 (81.6)
UICC 1	2 (5)
UICC 2	35 (87.5)
UICC 3	0
UICC 4	3 (7.5)
Control group	9 (18.4)
Resection margin	
R 0	9
R 1	31
Tumor marker	
CEA	1.4 (0.2 – 141.7)
CA19-9	78.3 (4.1 – 21078.4)

UICC – Union international contre le cancer; CEA – carcinoembryonic antigen; CA 19-9 – carbohydrate antigen 19-9.

Postoperative morbidity occurred in 9 patients (22%) with pancreatic cancer; 5 of these patients had a pancreatic fistula. The in-hospital mortality was 2.4% (*n* = 1).

Patients with a chronic pancreatitis received a duodenum-preserving pancreatic head resection.

### Expression of serum growth factors in PDAC and chronic pancreatitis

Different growth factors in patients with chronic pancreatitis and PDAC were compared preoperatively. In patients with PDAC, serum levels of FGF (*P* = 0.002), G-CSF (*P* = 0.037) and TNF-α (*P* = 0.044) were significantly decreased, compared to patients with chronic pancreatitis (Table 2).

**Table 2:** Comparison of circulating angiogenic cytokines in patients with pancreatic adenocarcinoma and a benign pancreatic disease (preoperatively)

	Pancreatic cancer (*n* = 40)	Control Group (*n* = 9)	*p*-value
	Median (range)	Median (range)	
**EGF**	370.2 (25.2–876.8)	386.3 (187.4–705.2)	0.68
**Angio 1**	38111 (1527–64586)	32264 (22282–46873)	0.61
**IL-4**	8.8 (4.0–24.5)	11.8 (6.0–46.5)	0.056
**IL-6**	19.0 (4.0–116.5)	20.0 (7.0–85.0)	0.51
**IL-8**	23.3 (0–346.2)	15.4 (0–19.6)	0.17
**PDGF-AA**	2755.7 (153.7–5465.4)	2493.8 (1399.5–3459.8)	0.64
**PlGF**	14.5 (7.5–28.6)	13.1 (7.5–16.4)	0.2
**FGF**	21.5 (5.0–66.5)	43.5 (10.0–100.8)	**0.002**
**G-CSF**	14.5 (5.0–28.5)	16.8 (7.0–62.3)	**0.037**
**TNFα**	9.0 (3.0–98.5)	20.3 (5.0–78.3)	**0.044**
**VEGF**	140.8 (8.5–796.8)	165.0 (54.0–885.3)	0.29
**HGF**	303.0 (11.5–1155.8)	513.8 (219.5–763.8)	0.25
**SDF-1α**	36.5 (4.0–121.8)	55.3 (15.0–142.5)	0.22

EGF = epidermal growth factor, Ang-1 = angiopoetin-1, IL-4 = interleukin 4, IL-6 = interleukin 6, IL-8 = interleukin 8, PDGF-AA = platelet-derived endothelial growth factors-AA, PlGF = placental growth factor, FGF-basic = basic fibroblast growth factor, G-CSF = granulocyte colony stimulating factor, TNF- α = tumor necrosis factor alpha, VEGF = vascular endothelial growth factor, HGF = hepatocyte growth factor, SDF-1α = stromal cell-derived factor 1 alpha.

The comparison was also performed on POD3 and POD7. On POD3, there were no significant differences between PDAC patients and patients with chronic pancreatitis (Supplementary Table 1). One week postoperatively, the comparison revealed significantly lower levels of IL-4 (*P* = 0.016), FGF (*P* = 0.005), TNF-α (*P* = 0.033) and HGF (*P* = 0.024) in PDAC patients (Supplementary Table 2).

Since there were significantly different cytokine levels between patients with pancreatic cancer and chronic pancreatitis, only patients with pancreatic cancer were included in the following analysis.

### Expression of serum growth factors with preoperative clinicopathological parameters

In a further analysis, we evaluated whether CAC levels were associated with clinical and pathological variables of patients with PDAC. Gender did not show any correlation with serum levels of CAC, except for significantly higher levels of EGF in males (median 459, range 138–877 vs. female 316, 25–553; *p* = 0.002). Age (< 65 vs. > 65 years); T, N, or UICC stage did not have an influence on the cytokine levels. Furthermore, there was no difference in patients with or without postoperative morbidity.

### C reactive protein (CRP) and CACs

One week after the surgery, CRP was significantly lower than before surgery (118.9 ± 42.9 vs. 65.8 ± 50.3, *P* < 0.001). We analyzed the correlation between CRP and CACs before and a week after surgery. The only significant correlation was found between CRP and IL-6 post surgery (correlation coefficient = 0.647, *P* < 0.001).

### Correlation analysis of serum growth factors in patients with PDAC

Correlation analyses were performed to assess potential interactions of individual serum growth factors in patients with PDAC. Preoperative serum levels of EGF were correlated with those of PDGFA-A (*r* = 0.482), Ang-1 (*r* = 0.446) and HGF (*r* = 0.395). Furthermore, there were relevant correlations between circulating levels of PDGF-A and Ang-1 (*r* = 0.586) and VEGF (*r* = 0.738). PlGF was correlated with IL-6 (*r* = 0.497), IL-8 (*r* = 0.536), FGF (*r* = 0.49), G-CSF (*r* = 0.505), VEGF (*r* = 0.738), HGF (*r* = 0.479) and SDF-1α (*r* = 0.552). We also found a positive correlation of VEGF with IL-6 (*r* = 0.457), FGF (*r* = 0.44), G-CSF (*r* = 0.543), HGF (*r* = 0.586) and SDF-1α (0.784). Circulating levels of TNF-α correlated with IL-4 (*r* = 0.656), IL-6 (*r* = 0.822), FGF (*r* = 446), G-CSF (*r* = 0.529) and SDF-1α (*r* = 0.602) (Supplementary Table 3). Most of these correlations were also significantly evident on POD3 (Supplementary Table 4) and POD7 (Supplementary Table 5).

### Changes of CAC levels after pancreatic resection

Some cytokine levels significantly changed after pancreatic resection, whereas others remained at the same level. The value of EGF first drops to 72.3 % (*P* = 0.0001) on POD 3, before it starts rising again to 81.4 % (*P* = 0.02) on POD 7. Ang-1 levels also fall to 19.2 % (*P* = 0.02) on POD 3, before increasing to 62.5 % on POD 7. Other cytokines, e.g. PlGF, rose significantly after the operation (30.9 %; *P* = 0.0001) on POD 3, but their level did not change afterwards. Most of the cytokines were continuously ascending after the operation (IL-4, G-CSF, VEGF, SDF-1α) (Table 3).

**Table 3:** Changes of the cytokine parameters on the different postoperative days in PDAC

	Pre-operative	POD 3	*p*-value	POD 7	*p*-value
	Median (range)	Median (range)	(pre-OP vs. POD 3)	Median (range)	(POD 3 vs. 7)
**EGF**	370.2 (25–877)	100.4 (19– 937)	**0.0001**	182 (14.2–868.1)	**0.02**
**Angio 1**	38111 (1527–64568)	29557 (1533–72820)	**0.02**	50226 (148–96336)	**0.0001**
**IL-4**	8.8 (4–24.5)	9.2 (4.5–36)	0.18	11.5 (5.5–35.5)	0.32
**IL-6**	19 (4–116.5)	49.4 (5.5–1674)	0.06	35.3 (5.5–830)	0.13
**IL-8**	23.3 (0–346)	32.1 (9.9–172.8)	0.98	32.3 (16.4–379)	0.07
**PDGF-AA**	2756 (154–5465)	2664 (245–6189)	0.41	4106 (689–8844)	**0.0001**
**PlGF**	14.5 (7.5–28.6)	18.3 (10.7–33.7)	**0.0001**	19.4 (10.4–47.7)	0.46
**FGF**	21.5 (5–66.5)	19.5 (4–79.5)	0.77	30.5 (5.5–101.8)	**0.17**
**G-CSF**	14.5 (5–28.5)	16.8 (5–99.8)	**0.04**	21 (4.8–62.8)	0.94
**TNFα**	9 (3–98.5)	8 (2.5–163.3)	0.19	13.3 (2.8–38.5)	0.75
**VEGF**	140.8 (8.5–796.8)	193.3 (6.5–875)	0.15	298 (11–1726)	**0.01**
**HGF**	303 (11.5–1155)	471 (13.3–3235)	**0.006**	424.5 (20–1414)	0.1
**SDF-1α**	36.5 (4–121.8)	45.5 (3.5–161)	**0.02**	53.8 (4.5–193.3)	0.056

PDAC = pancreatic ductal adenocarcinoma, EGF = epidermal growth factor, Ang-1 = angiopoetin-1, IL-4 = interleukin 4, IL-6 = interleukin 6, IL-8 = interleukin 8, PDGF-AA = platelet-derived endothelial growth factors-AA, PlGF = placental growth factor, FGF-basic = basic fibroblast growth factor, G-CSF = granulocyte colony stimulating factor, TNF- α = tumor necrosis factor alpha, VEGF = vascular endothelial growth factor, HGF = hepatocyte growth factor, SDF-1α = stromal cell-derived factor 1 alpha.

### Multivariate survival analysis

Overall survival for PDAC was 19 (3–39) months. 31 patients (75.6 %) had a recurrence and 24 (58.5 %) patients had a tumor-related death.

The comparison of the cytokines with the timeline was most important in the detection of the factors influencing long-term survival. For this purpose, we compared the preoperative cytokine levels with those collected postoperatively. A recursive elimination of insignificant factors was used to arrive at the final regression model. The postoperative changes of the 13 different cytokines of every single patient were correlated with their survival. This showed that, within the postoperative changes, increased levels of EGF (*P* = 0.023), PDGFA-A (*P* = 0.024), TNF-α (*P* = 0.001) and IL-8 (*P* = 0.049) in each patient were significantly correlated with improved survival, while elevated VEGF levels (*P* = 0.005) with impaired survival in the postoperative course (Table 4).

**Table 4:** Multivariate cox regression model comparing the changes of the cytokines pre and postoperatively

	Coef	Exp (coef)	Se (coef)	z	*p*-value
**EGF**	−0.003	0.997	0.001	−2.28	0.023
**PDGF-AA**	−0.0006	0.999	0.0003	−2.27	0.024
**TNF-α**	−0.125	0.882	0.04	−3.12	0.001
**VEGF**	0.011	1.011	0.004	2.79	0.005
**IL8**	−0.019	0.98	0.01	−1.97	0.049

EGF = epidermal growth factor, PDGF-AA = platelet-derived endothelial growth factors-AA, TNF- α = tumor necrosis factor alpha, VEGF = vascular endothelial growth factor, IL-8 = interleukin 8.

## DISCUSSION

The present study analyzed the postoperative course of CACs in patients who underwent elective pancreatic resection for PDAC or CP. The findings demonstrate that the expression of CACs in patients with PDAC differs from those with CP both pre- and postoperatively. For the first time, we were able to demonstrate the postoperative changes of 13 different cytokines and their interactions in the same patient cohort. Furthermore, in a multivariate analysis, we could show that the postoperative changes of five cytokines had an influence on long-term survival.

### Differences between PDAC and CP

Epidemiological studies and clinical observations concluded that patients with CP have an increased risk for developing PDAC [22]. Many of the growth-promoting factors involved in tissue remodeling and regeneration in CP are often up-regulated in PDAC [23, 24]. However, data on comparison of their serum levels between CP and PDAC, before and after surgical resection, are very limited.

In our study, preoperative serum levels of FGF, G-CSF and TNF-α were significantly lower in patients with PDAC, compared with CP. One week after surgery, we observed lower serum levels of IL-4, FGF, TNF-α and HGF in patients with PDAC than in patients with CP. These findings are not completely in agreement with previous studies: Talar-Wojnarowska et al. reported serum levels of TNF-α to be significantly higher in PDAC patients compared with CP patients [25]. Several reasons may be responsible for the controversy. First of all, our findings are observed in sera of patients with PDAC and CP. In the majority of previous studies, however, angiogenic factors have been quantified in tumor tissues [26, 27]. Secondly, since we aim to observe the postoperative course of CACs, we included a clearly defined group, namely patients with curative resectable PDAC. The contrary findings may be partially attributed to the diversity of patient populations [28]. In the study by Talar-Wojnarowska et al., 78 % of included patients did not undergo a curative resection and received only palliative surgery, chemotherapy or endoscopic treatment [25].

### Correlation between the CACs

Although a compelling body of evidence indicates that tumor angiogenesis is governed by various countervailing factors in solid human malignancies [29, 30], little is known about the interplay of these factors, in particular with respect to PDAC.

Our analyses show multiple correlations between the 13 different CACs preoperatively as well as postoperatively. For example, TNF-α was correlated with serum IL-4 at all of the three measurement points of time. Furthermore, none of the analyzed CAC correlated with patients’ prognosis on univariate analyses. This notion is supported by findings in esophageal cancer [31].

Due to the central role of angiogenesis for the growth and metastatic progression of tumors, angiogenic cytokines have been investigated intensively as targets for systemic therapy. However, resulting long-term clinical responses have been disappointing [8, 19, 20]. Angiogenic factors may also serve as biomarkers to predict the response or resistance to chemotherapy or anti-angiogenic therapy. To date, however, there are few validated biological markers to select cancer patients accurately for systemic therapy. One possible explanation is that the majority of available studies were limited to expression and biological function of a single or very few angiogenic factors, curbing conclusions about the complex network of different angiogenic factors. Kopetz et al. examined changes of various circulating cytokines in 43 patients receiving anti-angiogenic therapy with bevacizumab for metastatic colorectal cancer and found several of these factors to be increased prior to radiological development of progressive disease [32]. Together with these data, our findings suggest that the lack of one single therapeutic target or predictive biomarker for tumor therapy may be due to involvement of various angiogenic factors and the complex interaction. Biological functions of CACs and their usefulness as prognostic factors in clinical practice should not be considered separately. Thus, the simultaneous analysis of a whole cluster of CACs is meaningful and necessary.

CRP is a typical marker of inflammation, which can be evaluated in a non-invasive manner. The correlation between CRP and CACs before and a week after surgery was also analyzed. The only significant correlation was found between CRP and IL-6 post surgery. This result is not surprising, since IL-6 is known as a pro-inflammatory cytokine and a main driver of CRP [33]. These data imply that the postoperative changes of CACs were not totally driven by inflammatory processes.

### Postoperative course of CACs and prognosis

Because of the central role of angiogenesis for the growth and metastatic progression of tumors, angiogenic cytokines have been investigated intensively as potential markers for prognosis. In most of the existing studies, CACs were analyzed at one single moment (usually preoperatively) [24], which cannot represent the postoperative changes of these factors. To predict outcome after surgery, postoperative fluctuation of CAC levels should be considered. For the first time, we analyzed the postoperative course of 13 different CACs simultaneously and their influence on survival. We observed significant changes in the levels of several CACs. Owing to the differences in their biological function, a general tendency in the postoperative course cannot be detected. Within the postoperative changes, increasing levels of EGF, PDGFA-A, TNF-α and IL-8 in each patient were significantly correlated with improved survival, while decreasing VEGF levels with impaired survival in the postoperative course. Among the five cytokines, the correlation of VEGF and TNF-α and long-term survival is most significant.

The exact biological function of VEGF and its interaction with other angiogenic factors in pancreatic cancer remains poorly understood. The value of VEGF as a predictor of survival in patients with pancreatic cancer is controversial [16, 17]. Contradictory to other studies, we demonstrated an inverse correlation of circulation VEGF levels with survival. Unlike some of these studies, our data were confirmed by multivariate analysis with consideration of other angiogenic cytokines. This suggests that in PDAC, angiogenesis is not only driven by VEGF, but also by alternative signaling involving other cytokines. This theory is in line with the findings of Rahbari et al. [10] and is further supported by the fact that anti-VEGF therapy failed to demonstrate a permanent clinical response [20].

The present evaluation of serum TNF-α levels in patients who had undergone curative resection revealed a mild increase postoperatively, but the change did not reach statistical significance. In the multivariate analysis, ascending levels of TNF-α correlate with improved survival. TNF-α plays a paradoxical role in the evolution of cancer. TNF-α was first identified for its ability to induce rapid hemorrhagic necrosis of experimental cancers [34]. However, as the association between chronic inflammation and cancer is now appreciated, TNF-α, a master mediator of inflammation, enhances tumor development and spread [35]. A preclinical study showed that anti-TNF antibody inhibits pancreatic tumor growth and metastasis. Moreover, it reduces the size of recurrent tumor and diminishes the number of metastases after surgical resection [36]. However, TNF-α is merely weakly expressed by tumor cells. A retrospective analysis of 40 colorectal cancer patients revealed a potential beneficial effect of TNF-α by showing that increased TNF-α concentration in the sera correlated with improved survival [37]. Reissfelder et al. demonstrated that TNF-α acts as an indicator of tumor-specific cytotoxic T lymphocyte activity *in situ*, and that increased TNF-α concentration in colorectal cancer tissue is an independent factor of improved survival [38]. Thus, in contrast to tumor cell-derived TNF-α, immune cell- derived TNF-α may exert protective effects, or at least indicate protective antitumor immune responses. This is consistent with our findings that a higher serum TNF- α level is strongly correlated with patients’ improved outcome.

The small number of the control group may be a limitation. A larger number and balanced data would be more meaningful to make a statistical comparison.

The present study analyzed the expression and biological relevance of 13 CACs in patients who had undergone curative pancreatic resection for PDAC. The results indicate that certain angiogenic cytokines are differently expressed in PDAC from CP. Postoperative changes of serum levels of five cytokines correlate with patients’ prognosis after resection for PDAC, if a panel of several CACs is considered simultaneously. TNF and VEGF had the strongest impact on survival. Based on these observations, further studies on the biological role and prognostic relevance of CACs should not be limited to single molecules. In addition, anti-angiogenic therapy in patients with pancreatic cancer should be multi-targeted.

## MATERIALS AND METHODS

Blood samples were taken of 63 patients with histologically confirmed pancreatic ductal adenocarcinoma (PDAC) who had undergone resection with curative intent in the Department of General, Visceral and Transplantation Surgery of the University of Heidelberg between 2009 and 2011. Patients with a history of a second malignancy or neoadjuvant chemotherapy/ chemoradiotherapy were excluded from the analysis. For further analysis, only patients with complete blood samples on all follow-up dates were included (*n* = 40). To evaluate the impact of surgical procedures as an inducer of growth factors, our study also enrolled nine patients who had undergone pancreatic resection for chronic pancreatitis. All patients gave written informed consent. The study protocol was approved by the Ethics Committee of the University of Heidelberg.

Tumor stage and grade were classified according to the 7th edition of the TNM classification of the UICC (International Union Against Cancer), and the entire population under study was stratified according to the UICC stages. R1 resection was defined if the resection margin was less than 1 mm. Adjuvant chemotherapy with gemcitabine was recommended to all patients who were in a good overall condition. The length of follow-up was calculated from the date of operation at our institution. All patients were entered in a prospectively maintained database, including data on morbidity, in-hospital mortality and a long-term follow up of at least 30 months. Postoperative follow-up was performed at our outpatient clinics, the European Pancreas Center (EPC) or by their primary care physicians. Follow-up visits were scheduled every three months in the first two years and six-monthly thereafter. Each follow-up visit included a clinical examination, abdominal ultrasound and routine laboratory testing with evaluation of carbohydrate antigen 19-9 (CA19-9) levels. In addition, a CT scan was performed at three months postoperatively and every 6 months thereafter. Occurrence of distant metastases and local recurrences, follow-up time and reason for death were obtained for each patient to assess disease-specific survival.

### Serum preparation and cytokine detection

Venous blood samples were taken preoperatively (on the day of surgery) and on Day 3 (POD 3) and Day 7 (POD 7) postoperatively. Blood was collected using serum separator tubes. The tubes were immediately centrifuged at 2500 g for 10 minutes and the separated serum was stored at −80°C. Serum concentrations of interleukin 4 (IL-4), interleukin 6 (IL-6), basic fibroblast growth factor (FGF-basic), granulocyte colony stimulating factor (G-CSF), tumor necrosis factor alpha (TNF-alpha), vascular endothelial growth factor (VEGF), hepatocyte growth factor (HGF) and stromal cell-derived factor 1 alpha (SDF-1α) were detected, using Multiplex Human Cytokine Assay (BioRad Laboratories, Hercules, CA, USA) and a two-laser array reader, according to the manufacturers’ protocol. Concentrations of growth factors were analyzed and calculated by Bio-Plex Manager 4.1.1.

Serum concentrations of interleukin 8 (IL-8), epidermal growth factor (EGF), angiopoetin-1 (Ang-1), platelet-derived endothelial growth factors (PDGF-AA) and placental growth factor (PlGF) were determined, using commercially available quantitative sandwich enzyme-linked immunosorbent assay (ELISA) kits (Quantikine; R&D Systems, Inc, Minneapolis, MN) and a microtiter plate reader (ELISA Reader 2010, Anthos Mikrosysteme GmbH, Krefeld, Germany), according to the manufacturers’ protocol. All samples were assayed in duplicate and the mean value was used for quantity determination.

### Statistical analyses

Distributions of continuous variables were described by their medians and interquartile ranges. Two group comparisons were done, using Wilcoxon's rank sum test for unpaired data and Wilcoxon's signed rank test for paired data. Categorical variables were characterized by counts and proportions and compared using Fisher's exact test. Results of experimental analyses were compared using paired and unpaired *t*-tests. Correlation analyses were performed based on Pearson's correlation coefficient. The proportional hazards regression model of Cox was used to investigate the influence of the cytokines on survival. A *p*-value < 5 % was considered as statistically significant. All analyses were performed using SPSS@ software version 21 (SPSS, Chicago, Illinois, USA).

## SUPPLEMENTARY MATERIALS TABLES


