# Supplementary material for: Postoperative course and prognostic value of circulating angiogenic cytokines after pancreatic cancer resection
Source: Oncotarget. 2017 Aug 17;8(42):72315–23. doi: 10.18632/oncotarget.20315 (PMC5641132; doi:10.18632/oncotarget.20315)
Supplement: Supplementary file 1 [file oncotarget-08-72315-s001.pdf]

Postoperative course and prognostic value of circulating angiogenic cytokines after pancreatic cancer resection

SUPPLEMENTARY MATERIALS

Supplementary Table 1: Comparison of circulating angiogenic cytokines in patients with pancreatic adenocarcinoma and a benign pancreatic disease on POD 3

	Pancreatic cancer	Chronic pancreatitis	*p*-value
	Median (range)	Median (range)	
**EGF**	100.43 (19–937)	61.26 (24–291)	0.77
**Angio 1**	29557 (1533.4 – 72820.0)	18531 (10757–40616.0)	0.054
**IL-4**	9.15 (4.5–36.0)	18.9 (7.5–25.8)	0.11
**IL-6**	49.4 (5.5–1674.3)	175.5 (54.3–278.5)	0.71
**IL-8**	32.11 (9.92–172.86)	28.19 (8.44 – 57.7)	0.4
**PDGF-AA**	2663.7 (245,3–6189.2)	1889.5 (1151.8–3403.7)	0.09
**PlGF**	18.28 (10.7–33.7)	19.20 (14.1–40.5)	0.34
**FGF**	19.5 (4.0–79.5)	35.65 (13.5–63.5)	0.19
**G-CSF**	16.8 (5.0–99.8)	27.5 (16.3–53.3)	0.58
**TNFα**	8 (2.5- 163.3)	16.65 (6.0–22.0)	0.99
**VEGF**	193.3 (6.5–874.8)	188.5 (92.3–749.8)	0.77
**HGF**	471.0 (13.3–3235.3)	576.9 (451–1010.5)	0.89
**SDF-1α**	45.5 (3.5–161.3)	56.75 (27.8–107.8)	0.77

EGF = epidermal growth factor, Ang-1 = angiopoetin-1, IL-4 = interleukin 4, IL-6 = interleukin 6, IL-8 = interleukin 8, PDGF-AA = platelet-derived endothelial growth factors-AA, PlGF = placental growth factor, FGF-basic = basic fibroblast growth factor, G-CSF = granulocyte colony stimulating factor, TNF- α = tumor necrosis factor alpha, VEGF = vascular endothelial growth factor, HGF = hepatocyte growth factor, SDF-1α = stromal cell-derived factor 1 alpha.

Supplementary Table 2: Comparison of circulating angiogenic cytokines in patients with pancreatic adenocarcinoma and a benign pancreatic disease (1 week postoperatively)

	Pancreatic cancer	Chronic pancreatitis	*p*-value
	Median (range)	Median (range)	
**EGF**	182,07 (14,2–868,1)	112,2 (33,3–820,4)	0,964
**Angio 1**	50226 (147,8–96336)	36529,5 (7381,4–72249,0)	0,110
**IL-4**	11,5 (5,5–35,5)	19,4 (9,5–60,6)	**0,016**
**IL-6**	35,3 (5,5–830)	84,2 (25,0–212,8)	0,699
**IL-8**	32,3 (16,4–379,2)	30,6 (17,6–151,8)	0,687
**PDGF-AA**	4105,7 (689,3–8844,4)	3908,4 (1095,0–7299,3)	0,500
**PlGF**	19,4 (10,4–47,7)	21,5 (9,1–37,0)	0,362
**FGF**	30,5 (5,5–101,8)	60,4 (33,8–146,3)	**0,005**
**G-CSF**	21,0(4,8–62,8)	26,5(15,3–118,0)	0,058
**TNFα**	13,3 (2,8–38,5)	21,0 (7,0–78,5)	**0,033**
**VEGF**	298,0 (11,0 –1725,8)	466,8 (112,5–2555,0)	0,087
**HGF**	424,5 (20,0–1414,3)	874,0 (233,0–1545,5)	**0,024**
**SDF-1α**	53,8 (4,5–193,3)	56,25 (22,8–185,8)	0,786

EGF = epidermal growth factor, Ang-1 = angiopoetin-1, IL-4 = interleukin 4, IL-6 = interleukin 6, IL-8 = interleukin 8, PDGF-AA = platelet-derived endothelial growth factors-AA, PlGF = placental growth factor, FGF-basic = basic fibroblast growth factor, G-CSF = granulocyte colony stimulating factor, TNF- α = tumor necrosis factor alpha, VEGF = vascular endothelial growth factor, HGF = hepatocyte growth factor, SDF-1α = stromal cell-derived factor 1 alpha.

Supplementary Table 3: Correlation of circulating angiogenic cytokines in patients with pancreatic adenocarcinoma (preoperatively)

	EFG	Angio 1	IL 8	PlGF	PDGF−AA	IL 4	IL 6	FGF	G−CSF	TNF−α	VEGF	HGF	SDF−1α
**EFG**	1	0.577**	0.09	0.208	0.468**	−0.031	−0.088	0.071	0.012	−0.012	0.218	0.395*	0.152
		0	0.6	0.225	0.004	0.873	0.65	0.713	0.95	0.949	0.256	0.034	0.432
**Angio 1**	0.577**	1	0.06	0.089	0.586**	0.179	0.138	0.178	0.18	0.27	0.217	0.201	0.316
	0		0.728	0.606	0	0.354	0.476	0.355	0.349	0.157	0.259	0.295	0.094
**IL 8**	0.09	0.06	1	0.536**	0.046	0.201	0.252	0.315	0.359	0.02	0.149	0.24	0.025
	0.6	0.728		0.001	0.79	0.296	0.188	0.096	0.056	0.917	0.441	0.211	0.899
**PlGF**	0.208	0.089	0.536**	1	0.173	0.296	0.497**	0.490**	0.505**	0.178	0.738**	0.479**	0.552**
	0.225	0.606	0.001		0.312	0.119	0.006	0.007	0.005	0.356	0	0.009	0.002
**PDGF-AA**	0.468**	0.586**	0.046	0.173	1	0.091	0.128	0.086	0.127	0.073	0.379*	0.362	0.289
	0.004	0	0.79	0.312		0.638	0.508	0.657	0.511	0.706	0.042	0.053	0.128
**IL 4**	−0.031	0.179	0.201	0.296	0.091	1	0.619**	0.587**	0.904**	0.656**	0.312	0.399*	0.534**
	0.873	0.354	0.296	0.119	0.638		0	0.001	0	0	0.087	0.026	0.002
**IL 6**	−0.088	0.138	0.252	0.497**	0.128	0.619**	1	0.441*	0.649**	0.822**	0.457**	0.311	0.677**
	0.65	0.476	0.188	0.006	0.508	0		0.013	0	0	0.01	0.089	0
**FGF**	0.071	0.178	0.315	0.490**	0.086	0.587**	0.441*	1	0.682**	0.446*	0.440*	0.373*	0.426*
	0.713	0.355	0.096	0.007	0.657	0.001	0.013		0	0.012	0.013	0.039	0.017
**G-CSF**	0.012	0.18	0.359	0.505**	0.127	0.904**	0.649**	0.682**	1	0.529**	0.543**	0.535**	0.660**
	0.95	0.349	0.056	0.005	0.511	0	0	0		0.002	0.002	0.002	0
**TNF-α**	−0.012	0.27	0.02	0.178	0.073	0.656**	0.822**	0.446*	0.529**	1	0.191	0.173	0.602**
	0.949	0.157	0.917	0.356	0.706	0	0	0.012	0.002		0.303	0.352	0
**VEGF**	0.218	0.217	0.149	0.738**	0.379*	0.312	0.457**	0.440*	0.543**	0.191	1	0.586**	0.784**
	0.256	0.259	0.441	0	0.042	0.087	0.01	0.013	0.002	0.303		0.001	0
**HGF**	0.395*	0.201	0.24	0.479**	0.362	0.399*	0.311	0.373*	0.535**	0.173	0.586**	1	0.550**
	0.034	0.295	0.211	0.009	0.053	0.026	0.089	0.039	0.002	0.352	0.001		0.001
**SDF-1α**	0.152	0.316	0.025	0.552**	0.289	0.534**	0.677**	0.426*	0.660**	0.602**	0.784**	0.550**	1
	0.432	0.094	0.899	0.002	0.128	0.002	0	0.017	0	0	0	0.001	

EGF = epidermal growth factor, Ang-1 = angiopoetin-1, IL-4 = interleukin 4, IL-6 = interleukin 6, IL-8 = interleukin 8, PDGF-AA = platelet-derived endothelial growth factors-AA, PlGF = placental growth factor, FGF-basic = basic fibroblast growth factor, G-CSF = granulocyte colony stimulating factor, TNF- α = tumor necrosis factor alpha, VEGF = vascular endothelial growth factor, HGF = hepatocyte growth factor, SDF-1α = stromal cell-derived factor 1 alpha.

The upper value indicates Spearman's correlation coefficient, whereas the lower value indicates the *p*- value (**p* < 0.05, ***p* < 0.01).

Supplementary Table 4: Correlation of circulating angiogenic cytokines in patients with PDAC on POD3

	EFG	Angio 1	IL 8	PlGF	PDGF−AA	IL 4	IL 6	FGF	G−CSF	TNF−α	VEGF	HGF	SDF−1α
**EFG**	1	**0.652****	−0.002	0.2	**0.595****	0.058	−0.103	0.208	0.018	−0.004	**0.413***	−0.034	0.201
		0	0.989	0.222	0	0.755	0.58	0.262	0.922	0.981	0.021	0.855	0.278
**Angio 1**	**0.652****	1	0.013	0.198	**0.680****	0.278	−0.081	0.324	0.071	0.245	**0.443***	−0.053	0.442*
	0		0.938	0.228	0	0.13	0.665	0.075	0.705	0.183	0.012	0.778	0.013
**IL 8**	−0.002	0.013	1	**0.407****	0.076	0.158	**0.653****	0.145	**0.396***	0.031	0.059	0.289	0.117
	0.989	0.938		0.009	0.647	0.395	0	0.437	0.027	0.87	0.753	0.114	0.531
**PlGF**	0.2	0.198	**0.407****	1	0.235	0.124	**0.655****	0.263	**0.589****	0.087	0.35	0.169	**0.385***
	0.222	0.228	0.009		0.149	0.506	0	0.153	0	0.641	0.054	0.363	0.033
**PDGF-AA**	**0.595****	**0.680****	0.076	0.235	1	0.207	−0.059	0.336	0.086	0.191	0.424*	0.074	0.353
	0	0	0.647	0.149		0.264	0.751	0.064	0.645	0.304	0.017	0.694	0.052
**IL 4**	0.058	0.278	0.158	0.124	0.207	1	0.094	**0.904****	**0.657****	0.776**	**0.655****	0.197	**0.711****
	0.755	0.13	0.395	0.506	0.264		0.609	0	0	0	0	0.28	0
**IL 6**	−0.103	−0.081	**0.653****	**0.655****	−0.059	0.094	1	0.083	0.721**	0.104	0.131	0.124	0.287
	0.58	0.665	0	0	0.751	0.609		0.652	0	0.572	0.476	0.5	0.111
**FGF**	0.208	0.324	0.145	0.263	0.336	**0.904****	0.083	1	**0.695****	**0.716****	**0.845****	0.293	**0.825****
	0.262	0.075	0.437	0.153	0.064	0	0.652		0	0	0	0.104	0
**G−CSF**	0.018	0.071	**0.396***	**0.589****	0.086	**0.657****	**0.721****	**0.695****	1	**0.534****	**0.628****	0.252	**0.710****
	0.922	0.705	0.027	0	0.645	0	0	0		0.002	0	0.165	0
**TNF−α**	−0.004	0.245	0.031	0.087	0.191	**0.776****	0.104	**0.716****	**0.534****	1	**0.602****	0.19	**0.722****
	0.981	0.183	0.87	0.641	0.304	0	0.572	0	0.002		0	0.297	0
**VEGF**	**0.413***	**0.443***	0.059	0.35	**0.424***	**0.655****	0.131	**0.845****	**0.628****	**0.602****	1	0.259	**0.868****
	0.021	0.012	0.753	0.054	0.017	0	0.476	0	0	0		0.152	0
**HGF**	−0.034	−0.053	0.289	0.169	0.074	0.197	0.124	0.293	0.252	0.19	0.259	1	0.271
	0.855	0.778	0.114	0.363	0.694	0.28	0.5	0.104	0.165	0.297	0.152		0.134
**SDF−1α**	0.201	**0.442***	0.117	**0.385***	0.353	**0.711****	0.287	**0.825****	**0.710****	**0.722****	**0.868****	0.271	1
	0.278	0.013	0.531	0.033	0.052	0	0.111	0	0	0	0	0.134	

PDAC = pancreatic ductal adenocarcinoma, EGF = epidermal growth factor, Ang-1 = angiopoetin-1, IL-4 = interleukin 4, IL-6 = interleukin 6, IL-8 = interleukin 8, PDGF-AA = platelet-derived endothelial growth factors-AA, PlGF = placental growth factor, FGF-basic= basic fibroblast growth factor, G-CSF = granulocyte colony stimulating factor, TNF-α = tumor necrosis factor alpha, VEGF = vascular endothelial growth factor, HGF = hepatocyte growth factor, SDF-1α = stromal cell-derived factor 1 alpha.
The upper value indicates Spearman's correlation coefficient, whereas the lower value indicates the p- value (*$p < 0.05$, **$p < 0.01$).

Supplementary Table 5: Correlation of circulating angiogenic cytokines in patients with pancreatic adenocarcinoma one week postoperatively

	EFG	Angio 1	IL 8	PlGF	PDGF-AA	IL 4	IL 6	FGF	G-CSF	TNF-α	VEGF	HGF	SDF-1α
**EFG**	1	**0.427****	–0.158	–0.254	0.18	0.32	–0.033	**0.376***	0.353	0.323	0.299	0.21	0.294
		0.007	0.335	0.118	0.274	0.085	0.862	0.04	0.056	0.082	0.109	0.264	0.114
**Angio 1**	**0.427****	1	–0.257	–0.245	**0.704****	**0.370***	–0.225	**0.393***	0.266	**0.373***	**0.413***	0.19	**0.424***
	0.007		0.115	0.133	0	0.044	0.231	0.032	0.155	0.042	0.023	0.315	0.019
**IL 8**	–0.158	–0.257	1	**0.738****	–0.311	0.025	**0.747****	–0.005	0.321	0.123	–0.119	0.298	–0.216
	0.335	0.115		0	0.054	0.894	0	0.977	0.084	0.516	0.532	0.11	0.252
**PlGF**	–0.254	–0.245	**0.738****	1	–0.249	0.135	0.319	–0.029	0.041	0.09	–0.072	0.048	–0.203
	0.118	0.133	0		0.126	0.476	0.086	0.878	0.831	0.638	0.704	0.799	0.281
**PDGF-AA**	0.18	**0.704****	–0.311	–0.249	1	0.113	–0.332	0.18	0.05	0.129	0.115	0.05	0.225
	0.274	0	0.054	0.126		0.552	0.073	0.341	0.794	0.499	0.547	0.792	0.233
**IL 4**	0.32	**0.370***	0.025	0.135	0.113	1	0.04	**0.850****	**0.717****	**0.865****	**0.576****	**0.467****	**0.522****
	0.085	0.044	0.894	0.476	0.552		0.831	0	0	0	0.001	0.008	0.003
**IL 6**	–0.033	–0.225	**0.747****	0.319	–0.332	0.04	1	0.029	**0.485****	0.114	0.09	0.35	–0.013
	0.862	0.231	0	0.086	0.073	0.831		0.878	0.006	0.542	0.63	0.054	0.945
**FGF**	0.376*	**0.393***	–0.005	–0.029	0.18	**0.850****	0.029	1	**0.815****	**0.936****	**0.715****	**0.557****	**0.731****
	0.04	0.032	0.977	0.878	0.341	0	0.878		0	0	0	0.001	0
**G-CSF**	0.353	0.266	0.321	0.041	0.05	**0.717****	**0.485****	**0.815****	1	**0.844****	**0.727****	**0.694****	**0.684****
	0.056	0.155	0.084	0.831	0.794	0	0.006	0		0	0	0	0
**TNF-α**	0.323	**0.373***	0.123	0.09	0.129	**0.865****	0.114	**0.936****	**0.844****	1	**0.662****	**0.552****	**0.656****
	0.082	0.042	0.516	0.638	0.499	0	0.542	0	0		0	0.001	0
**VEGF**	0.299	**0.413***	–0.119	–0.072	0.115	**0.576****	0.09	**0.715****	**0.727****	**0.662****	1	**0.669****	**0.905****
	0.109	0.023	0.532	0.704	0.547	0.001	0.63	0	0	0		0	0
**HGF**	0.21	0.19	0.298	0.048	0.05	**0.467****	0.35	**0.557****	**0.694****	**0.552****	**0.669****	1	**0.552****
	0.264	0.315	0.11	0.799	0.792	0.008	0.054	0.001	0	0.001	0		0.001
**SDF-1α**	0.294	**0.424***	–0.216	–0.203	0.225	**0.522****	–0.013	**0.731****	**0.684****	**0.656****	**0.905****	**0.552****	1
	0.114	0.019	0.252	0.281	0.233	0.003	0.945	0	0	0	0	0.001	

EGF= epidermal growth factor, Ang-1= angiopoetin-1, IL-4= interleukin 4, IL-6= interleukin 6, IL-8= interleukin 8, PDGF-AA= platelet-derived endothelial growth factors-AA, PlGF= placental growth factor, FGF-basic= basic fibroblast growth factor, G-CSF= granulocyte colony stimulating factor, TNF- α= tumor necrosis factor alpha, VEGF= vascular endothelial growth factor, HGF= hepatocyte growth factor, SDF-1α= stromal cell-derived factor 1 alpha.

The upper value indicates Spearman's correlation coefficient, whereas the lower value indicates the *p*- value (**p* < 0.05, ***p* < 0.01).
